# EDIII-Fc induces protective immune responses against the Zika virus in mice and rhesus macaque

**DOI:** 10.1371/journal.pntd.0011770

**Published:** 2023-11-20

**Authors:** Hailong Su, Jun Liu, Jianhai Yu, Zhenzhen Qiu, Wenhan Liang, Wangsheng Wu, Haifeng Mo, Hongwei Li, Wei Zhao, Weiwang Gu

**Affiliations:** 1 School of Laboratory Medicine and Biotechnology, Southern Medical University, Guangzhou, China; 2 Dermatology Hospital, Southern Medical University, Guangzhou, China; 3 BSL-3 Laboratory (Guangdong), Guangdong Provincial Key Laboratory of Tropical Disease Research, School of Public Health, Southern Medical University, Guangzhou, China; 4 Department of Hematologic Oncology, State Key Laboratory of Oncology in South China, Collaborative Innovation Center for Cancer Medicine, Sun Yat-sen University Cancer Center, Guangzhou, China; 5 Sun Yat-sen Memorial Hospital, Guangzhou, China; 6 Guangdong Provincial Key Laboratory of Large Animal Models for Biomedicine, South China Institute of Large Animal Models for Biomedicine, Wuyi University, Jiangmen, China; 7 Institute of Comparative Medicine & Laboratory Animal Center, Southern Medical University, Guangzhou, China; University of Pittsburgh, UNITED STATES

## Abstract

*Zika virus* can infect the fetus through the placental barrier, causing ZIKV congenital syndrome and even miscarriage, which can cause great harm to pregnant women and infants. Currently, there is no vaccine and drug available to combat the *Zika virus*. In this study, we designed a fusion protein named EDIII-Fc, including the EDIII region of Zika E protein and human IgG Fc fragment, and obtained 293T cells that stably secreted EDIII-Fc protein using the lentiviral expression system. Mice were immunized with the EDIII-Fc protein, and it was observed that viral replication was significantly inhibited in the immunized mice compared to non-immunized mice. In rhesus macaques, we found that EDIII-Fc effectively induce the secretion of neutralizing antibodies and T cell immunity. These experimental data provide valid data for further use of *Zika virus* E protein to prepare an effective, safe, affordable Zika vaccine.

## Author summary

*Zika virus* (ZIKV) is a member of the *Flavivirus* genus. ZIKV could be transmitted through mosquito bites. In the population, ZIKV can be transmitted through mother-to-child transmission, sexual transmission, and blood transmission. Clinical symptoms of ZIKV infection in adults include rash, fever, arthralgia, and conjunctivitis, and neurological symptoms in those with severe infection, manifesting as Guillain-Barre syndrome (GBS). ZIKV can infect the fetus through the placental barrier, causing ZIKV congenital syndrome and even miscarriage. ZIKV causes fatal damage to the central nervous system of infants. Due to the lack of effective drugs, vaccination is the main strategy to against ZIKV. Conventional vaccines against the Zika virus have the potential to cause antibody-dependent enhancement (ADE). In this study, the authors designed a fusion protein named EDIII-Fc, including the EDIII region of Zika E protein and human IgG Fc fragment. EDIII protein has been shown to be incapable of causing ADE. Mice and rhesus monkeys were immunized with EDIII-Fc. EDIII-Fc could induce protective antibodies in mice and rhesus monkeys and protect mice against the challenge of ZIKV. These experimental data provide valid data for further use of ZIKV E protein to prepare an effective, safe, affordable Zika vaccine.

## Introduction

*Zika virus* (ZIKV) is a member of the *Flavivirus* genus of the *Flaviviridae* family, which includes *Japanese encephalitis virus* (JEV), *Dengue virus* (DENV), *West Nile virus* (WNV), and *Yellow fever virus* (YFV) [1–4]. ZIKV was first isolated from febrile rhesus macaque sera at Zika forest in Uganda by Dick GW et al. in 1947. The virus was named after the location of its first discovery. In 1952, ZIKV was isolated from a human for the first time, confirming that the virus could infect humans [5,6].

ZIKV was initially transmitted through mosquito bites [7]. Animal studies have demonstrated that ZIKV-bearing Aedes Aegypti Mosquitoes can transmit the ZIKV to monkeys or mice through bites [8]. In the population, ZIKV can be transmitted through mother-to-child transmission, sexual transmission, and blood transmission [9]. Clinical symptoms of ZIKV infection in adults include rash, fever, arthralgia, and conjunctivitis [10,11], and neurological symptoms in those with severe infection, manifesting as Guillain-Barre syndrome (GBS) [12–15]. ZIKV can infect the fetus through the placental barrier, causing ZIKV congenital syndrome and even miscarriage [16,17]. ZIKV infection causes fatal damage to the central nervous system of infants [18].

ZIKV is a positive-stranded single-stranded RNA virus (+ssRNA) [19]. The open reading frame of ZIKV encodes three structural proteins and seven non-structural proteins; the structural proteins include envelope protein (E), capsid protein (C), and membrane protein (M), which are the key components of the viral particle [20]; seven non-structural proteins include NS1, NS2A, NS2B, NS3, NS4A, NS4B, and NS5 [21].

As a member of the genus Flavivirus, ZIKV is similar to the dengue virus in that it has an icosahedral spherical structure, with each asymmetric structure containing three parallel arranged envelope proteins, each of which forms a homodimeric structure [22]. The E protein consists of approximately 505 amino acids, with the extracellular region containing 406 amino acids [22,23]. On the viral surface, the extracellular domain of the E protein is attached to the C’-end stem region with an anchor region and anchored to the viral membrane [24].

The E protein mediates viral-cell fusion and is the major antigenic protein [25]. The extracellular domain of the E protein consists of three structural domains, the first structural domain (domain I, EDI), the second structural domain (EDII) and the third structural domain (EDIII). The EDI and EDII regions are interspersed and contain the 297 amino acids at the N terminus, with EDI containing amino acids 1–52, 132–192, 280–296 and EDII containing amino acids 53–131, 193–279; the EDIII region is relatively independent and contains the 297–406 amino acids at the C terminus of the extracellular domain [26,27]. The viral binding receptors to the cell are located in the EDIII region [20,28].

Due to the lack of effective drugs, vaccination is the main strategy to against ZIKV. Currently, various vaccine designs targeting the ZIKV are being explored, including live attenuated vaccines [29], inactivated vaccines [30], viral vector vaccines [31], peptide vaccines [32], subunit vaccines [33], and nucleic acid vaccines [34].

Among these approaches, subunit protein vaccines focusing on the Zika E protein, the main antigenic protein, have shown promising results in research studies [33,35,36].

However, it is important to note that neutralizing antibodies induced by the full-length dengue E protein can neutralize the virus but may also increase the risk of antibody-dependent enhancement (ADE) when they are in a sub-neutralized state [37]. The ADE mechanism has the potential to elevate the risk of severe disease during secondary flavivirus infections, complicating the development of flavivirus vaccines [38,39]. Studies on dengue virus have revealed that severe illnesses, such as dengue fever and heat shock induced by ADE, not only pose treatment challenges but also increase the likelihood of fatalities in infected individuals [37,40,41]. In contrast, utilizing only the EDIII region of the ZIKV E protein as an antigen can effectively reduce the risk of ADE [42].

In this study, we designed a fusion protein named EDIII-Fc, including the EDIII region of Zika E protein and human IgG Fc fragment, and obtained 293T cells that stably secreted EDIII-Fc protein using the lentiviral expression system. Subsequently, the immunogenicity of the EDIII-Fc protein was evaluated in both mice and Rhesus macaques.

## Results

### A 293T cell line that exhibited stable expression and secretion of the EDIII-Fc protein was successfully established

**Fig 1:**
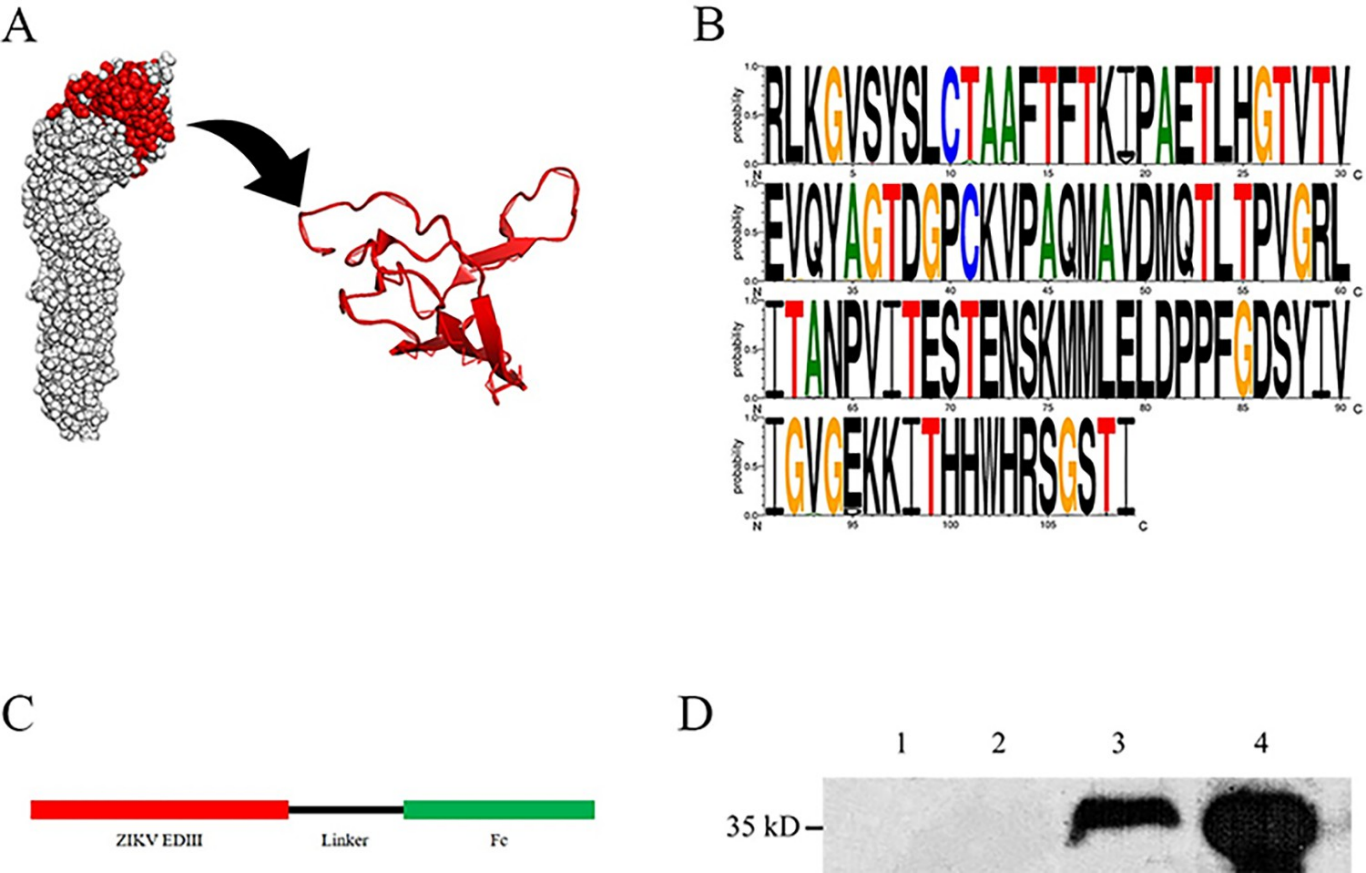
Expression of the recombinant EDIII-Fc protein. A. Positions of EDIII protein in the E protein. The crystal structure of the ZIKV E protein dimer (PDB ID 5LBV) is shown, with EDIII of monomeric subunit A colored in red. B. Sequence Logo visualization of the EDIII alignments. Sequence Logos of the EDIII domain from multiple sequence alignments across all organisms, consisting of 1,809 protein sequences (Sequence data from Genbank, the date to obtain the sequence was to June 20, 2023). C. Schematic map of pLV-ZIKV-EDIII vector construction. D. Western blot detection EDIII expression. Line 1, cell lysate of HEK293T transfected with pLV-eGFP; Line 2, supernatant of HEK293T transfected with pLV-eGFP; Line 3, cell lysate of HEK293T transfected with pLV-ZIKV-EDIII; Line 4, supernatant of HEK293T transfected with pLV-ZIKV-EDIII.

The EDIII structural domain of the ZIKV E protein is the target of effective neutralizing antibodies [43], and it consists of an IgG-like folded structure with a ring that combines several folded sheets with disordered chains (Fig 1A) [23]. The EDIII protein sequences derived from different hosts in the GenBank database were analyzed, and they showed above 96% identity (Figs 1B and S1). To synthesize the gene, we utilized the Haiti2014 strain sequence as a template and fused the EDIII sequence with human IgG Fc fragments to enhance its expression efficiency and immunogenicity (Fig 1C). By employing the lentiviral expression system, we successfully established a 293T cell line that exhibited stable expression and secretion of the EDIII-Fc protein. Western blot analysis confirmed predominant secretion of the recombinant EDIII-Fc protein (Fig 1D).

### Immunization with EDIII-Fc protects mice from ZIKV challenge

**Fig 2:**
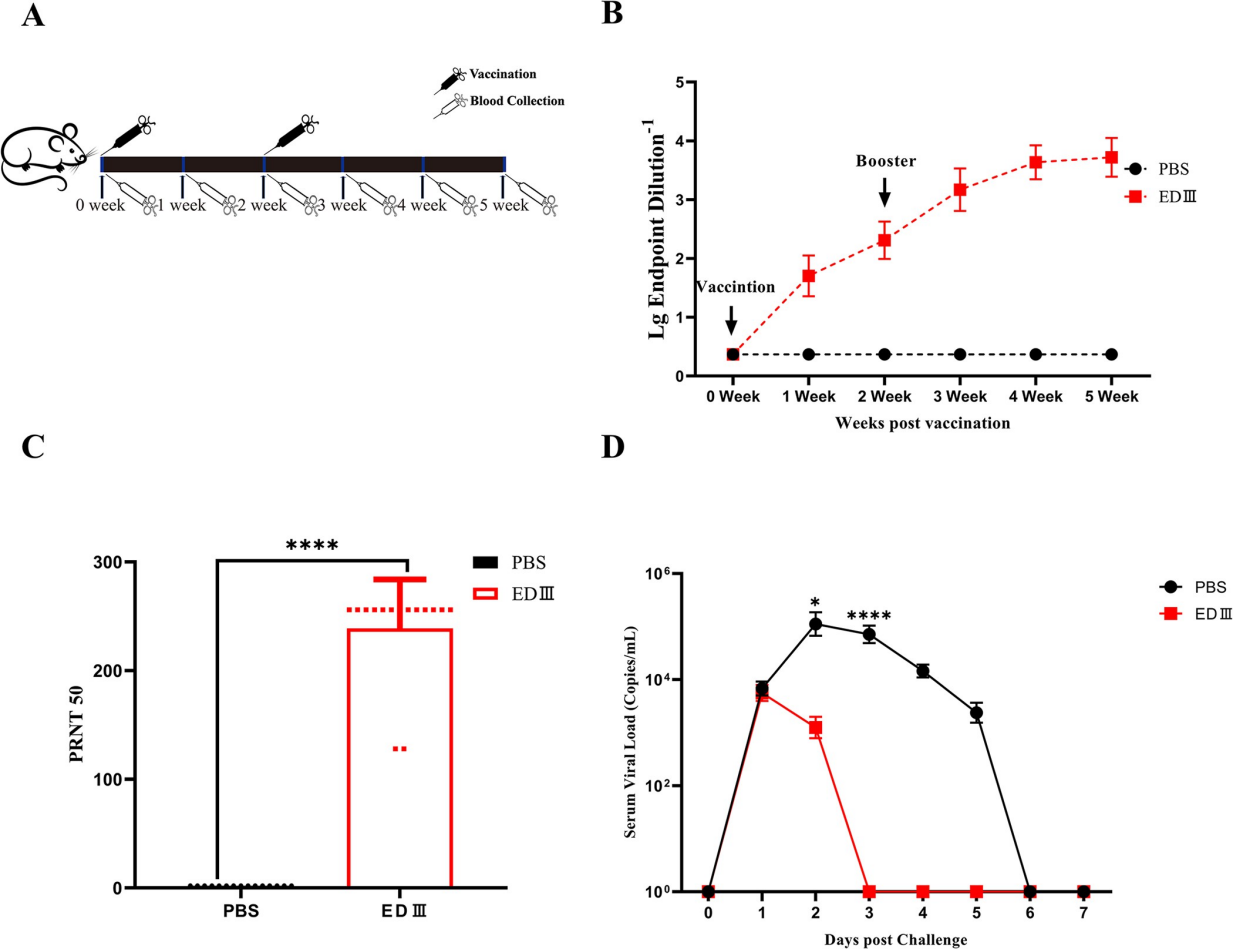
Immunization with EDIII-Fc protects mice from ZIKV challenge. A. Timeline for mice immunization with EDIII-Fc and blood collection. Balb/c mice were immunized with EDIII-Fc or PBS. Pre-immune blood samples were obtained 2 days before immunization, and subsequent samples were collected weekly post-immunization. B. Changes of total antibody levels in mice after immunization. Serum samples from mice immunized with EDIII-Fc (in red) or PBS (in black) were serially diluted at a 1:10 ratio. HRP-labeled sheep anti-mouse monoclonal antibody was used for ELISA detection. C. Detection of neutralizing antibody levels in mice. Two weeks after the second immunization, the sera to be tested were serially diluted 2-fold in PBS, starting from a 1:5 ratio, for plaque reduction neutralization testing. Results are reported as the fold dilution of serum that inhibited 50% of plaque formation. D. Serum viral load in mice after challenge. One week after the second immunization, seven Balb/c mice were injected subcutaneously with 10^4^ PFU of ZIKV. Daily blood samples were collected and qPCR was carried out for viral load detection. Three replications were done in each experiment, and the experiment was repeated three times. *: *P* < 0.05;****: *P* < 0.0001. Clipart image from https://openclipart.org/.

To assess the immunogenicity of the recombinant EDIII-Fc protein, the protein was combined with Freund’s complete adjuvant to create a vaccine. Balb/c mice were then subcutaneously immunized at multiple points in the abdominal region. The second and third immunizations were administered at 2 and 4 weeks, respectively, after the initial immunization (Fig 2A). Blood samples were collected weekly by tail amputation, and serum was prepared. ELISA results revealed that the total antibody titer was 1:100 in mice after the first immunization, which increased to 1:1000 following the booster immunization (Fig 2B). The plaque reduction neutralization assay demonstrated that the serum neutralizing antibody potency was 1:160 in mice after the booster immunization, showing high significance compared to the control group (Fig 2C, *P*<0.0001). On the 7th day after the second immunization, mice were subcutaneously injected with 10^4^ PFU ZIKV, and blood samples were collected daily thereafter to detect the ZIKV load in the serum. The viral load in the serum of vaccinated mice significantly decreased on the second day (*P*<0.05), and by the third day, ZIKV was undetectable (Fig 2D, *P*<0.0001). In contrast, control mice exhibited high levels of viral nucleic acid from days 1 to 5 after the challenge, with viral particles being cleared on day 6 (Fig 2D). These findings indicate that the EDIII-Fc protein can stimulate mice to produce elevated levels of antibodies that effectively neutralize ZIKV, both in *in vivo* and *in vitro* experiments. Moreover, immunization with the EDIII-Fc protein proves to be an effective measure in preventing ZIKV infection.

### EDIII -Fc induces the production of anti-ZIKV neutralizing antibodies in rhesus macaque

**Fig 3:**
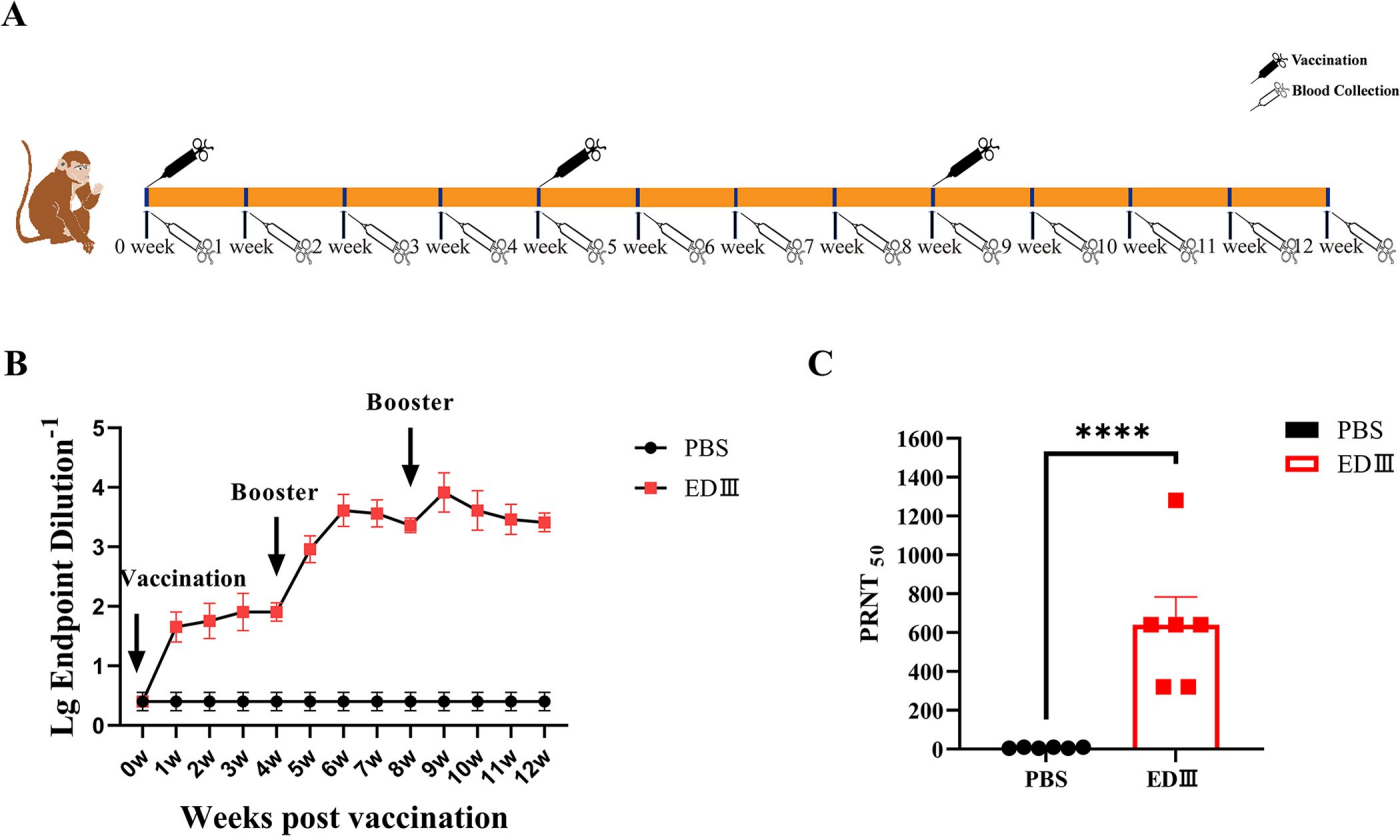
EDIII -Fc induction the production of anti-zika antibodies in rhesus macaques A. Timeline for rhesus macaque immunization with EDIII-Fc and blood collection. Rhesus macaques were immunized with EDIII-Fc or PBS. Pre-immune blood samples were obtained 2 days before immunization, and subsequent samples were collected weekly post-immunization. B. Changes of total antibody level in rhesus macaques after immunization. Serum samples from rhesus macaques immunized with EDIII-Fc (in red) or PBS (in black) were serially diluted at a 1:10 ratio. HRP-labeled mouse anti-monkey monoclonal antibody was used for ELISA detection. C. Detection of neutralizing antibody level in rhesus macaques. Two weeks after the third immunization, the sera to be tested were serially diluted 2-fold in PBS starting from 1:5 for plaque reduction neutralization test. Results are reported as the fold dilution of serum that inhibited 50% of plaque formation. Three replications were done in each experiment, and the experiment was repeated three times. ***: *P* < 0.0001. Clipart image from https://openclipart.org/.

To evaluate whether the EDIII-Fc protein can induce the production of protective antibodies in primates, this study used MF59 adjuvant-emulsified recombinant protein and immunized rhesus macaques (Fig 3A) by intramuscular injection. The level of EDIII-Fc protein-specific antibodies was measured by ELISA at the second week after the first immunization, and the total antibody titer of serum in the subunit vaccine group reached 1:300, which was significantly higher than that in the control group. The antibody titer reached 1:20,000 at the second week after the third immunization (Fig 3B). The results of the plaque reduction neutralization assay with ZIKV showed that the serum neutralizing antibody potency was 1:640 in rhesus macaques four weeks after the third immunization (Fig 3C), which was significantly higher than that in the control group. These results indicate that, similar to the mouse results, the EDIII protein induces high levels of anti-ZIKV neutralizing antibody production in rhesus macaques.

### EDIII induces T-cell immunity against ZIKV in rhesus macaques

**Fig 4:**
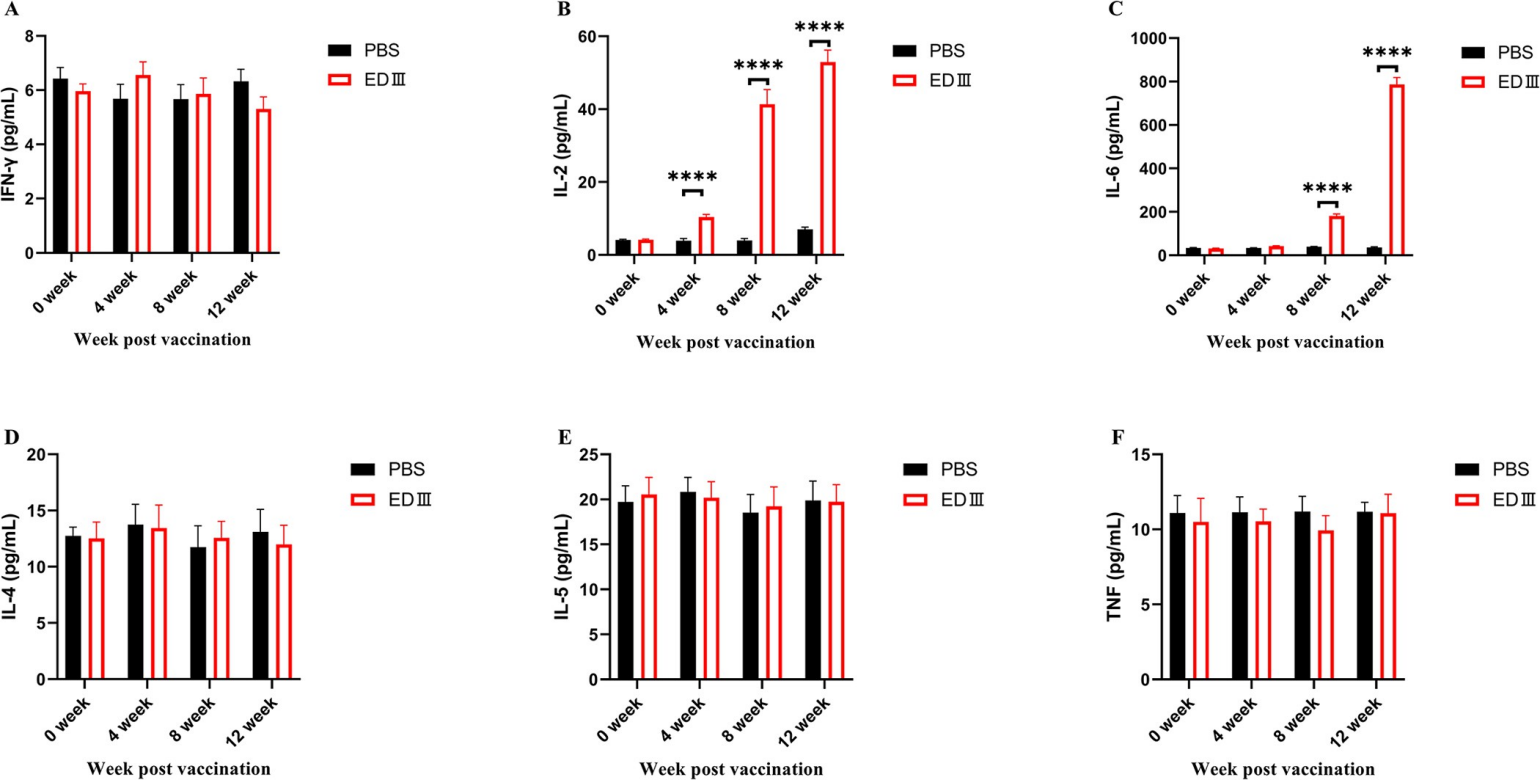
Changes in cytokines levels secreted in rhesus macaque after immunization. Pre-immune PBMCs and those obtained at 4 weeks, 8 weeks, and 12 weeks after immunization from rhesus macaques were incubated with ZIKV E protein for 48 hours. Following incubation, culture supernatants were collected for flow cytometric assays. Three replications were done in each experiment and repeated three times. ****: *P* < 0.0001.

Whole blood from the monkeys were collected, and peripheral blood mononuclear cells were isolated, and cultured in 1640 medium containing EDIII. We then measured the secretion of cytokines in the supernatant. Th1 cells mainly secrete IFN-γ and IL-2. The content of IFN-γ and IL-2 secreted by peripheral blood mononuclear cells in the control group did not change significantly compared to before immunization (IFN-γ, *P* = 0.0773; IL-2, *P* = 0.0584, Fig 4A and 4B). The IL-2 content in the culture supernatant of the subunit group was significantly higher than the level before immunization and the control group in the fourth week after the third immunization, while the IFN-γ content was not significantly different. Th2 cells mainly secrete IL-4, IL-5, and IL-6. The results showed that the serum IL-6 level was significantly higher in the subunit group than in the control group in the fourth week after the second immunization (172.533±22.186 pg/mL vs. 25.133±8.532 pg/mL, *P* = 0.0027, Fig 4C). The concentration of IL-6 in the cell culture supernatant of the subunit vaccine group reached 877.633±152.693 pg/mL in the fourth week after the third immunization. However, the differences in IL-4 and IL-5 levels between the subunit and control groups after three immunizations were insignificant (IL-4, *P* = 0.073; IL-5, *P* = 0.086, Fig 4D and 4E). The cells that secrete TNF mainly include activated macrophages, T cells, and epithelial cells. The results showed that the TNF content in the peripheral blood mononuclear cell culture supernatant of rhesus macaque in the subunit vaccine group in the fourth week after triple immunization was not significantly different from that of the control group (*P* = 0.1473, Fig 4F). These results indicate that EDIII-Fc induces the production of T-cell immunity against ZIKV.

## Discussion

Approximately 2 billion people worldwide live in tropical and subtropical regions where the ZIKV poses a threat, and with increased globalization, the risk of Zika transmission continues to rise [44,45].Therefore, there is an urgent need for a safe and effective Zika. In recent years, with the development of vaccine technology, scientists from different vaccine countries have been developing Zika vaccines through different technical routes. Inactivated whole-virus vaccines and live ZIKV have been shown to induce protective immune responses in mice [46]. However, it should be noted that antibodies induced by full-length E protein have the potential to cause ADE *in vivo* [47]. Nucleic acid vaccines and viral vector vaccines have also emerged as effective means of protecting against ZIKV infection in both mice and rhesus macaques [48–50]. Subunit vaccines offer several advantages over other vaccine types, including higher safety, better stability, long-lasting immunization, and cost-effectiveness.

Flaviviruses share a high degree of structural similarity [51,52]. The envelope protein of flaviviruses serves as the primary antigen on the virus’s surface, eliciting immunologic responses in infected hosts [53]. *Dengue virus* (DENV) and *Zika virus* (ZIKV) exhibit a significant level of homology in their envelope (E) proteins, with a similarity exceeding 50% [54,55]. Antibody-Dependent Enhancement (ADE) can be triggered by primary infection or by antibodies produced during vaccination. These antibodies cross-react with closely related flaviviruses but have limited neutralizing capacity [47]. Epidemiological analysis has observed that Zika virus outbreaks primarily occur in regions where Dengue virus is endemic. Additionally, there is a correlation between the severity of ZIKV infection and the presence of pre-existing DENV antibodies before ZIKV infection [47]. Studies conducted both in vitro and in vivo (using mice) have demonstrated that antibodies targeting either DENV or ZIKV in human serum can induce ADE for either ZIKV or DENV [56,57]. Individuals with a history of ZIKV infection are at an elevated risk of developing symptomatic DENV infections upon subsequent exposure, highlighting the potential for ZIKV-induced antibodies to exacerbate DENV infections [57]. Although the precise mechanisms are still under investigation, it is increasingly evident that prior immunity to ZIKV may, in certain cases, lead to an increased susceptibility to severe manifestations of DENV infection. Consequently, in the development of vaccines for DENV and ZIKV, careful consideration should be given to preventing the induction of ADE.

During the study of the dengue virus, which is also a flavivirus, it was found that inactivated dengue virus induced ADE of infection [58]. In contrast, the dengue E protein’s third structural domain is the main receptor binding region of the dengue virus and is effective in reducing the ADE effect [42]. The E protein third structural domain (EDIII) of ZIKV is similar to that of the dengue virus, is the major receptor binding region, and induces protective neutralizing antibodies in mice [59]. Monoclonal antibodies that target the epitope on the lateral ridge of the ZIKV envelope domain III (EDIII) have been found to provide effective protection in both mice and rhesus macaques [60,61]. Furthermore, antibodies induced by subunit vaccines that are based on the ZIKV EDIII protein have been demonstrated to be incapable of causing ADE [62–64].

In recent reports on EDIII protein-induced immunity in mice, some investigators have concluded that EDIII protein is not capable of inducing higher neutralizing antibodies in mice [65–67]. However, it has been shown that even at lower neutralizing titers, the EDIII protein can still produce protective effects in mice [66]. After three immunizations, Virus-Like-Particle (VLP) based EDIII without adjuvant could induce virus-neutralizing antibodies with a titer of 1:10. However, when administered with adjuvant, the titer of virus-neutralizing antibodies could reach 1:100 [67]. To enhance the immunogenicity and improve the soluble expression of the vaccine, as well as prolong its half-life in vivo, a fusion protein was developed by selecting the third structural domain (EDIII) of the ZIKV E protein and fusing it to a human Fc fragment [61,68]. The immunogenicity of this EDIII-Fc protein was assessed in both mice and rhesus macaques. Following the secondary immunization, mice exhibited neutralizing antibody titers of up to 1:160 two weeks after the second immunization (Fig 2C). Similarly, rhesus macaques demonstrated neutralizing antibody titers of 1:640 two weeks after the triple immunization (Fig 3C). These findings demonstrate that immunization with the recombinant EDIII-Fc protein triggers an antigen-specific response, leading to the production of neutralizing antibodies against ZIKV. Importantly, the observed ZIKV neutralization titers have been correlated with protection against ZIKV challenge in mice.

T cell immunity plays a crucial role in the defense against viral infections, working synergistically with B lymphocytes to clear viral particles in vivo. Previous studies have demonstrated that vaccine designs incorporating the full-length E protein successfully induce protective neutralizing antibodies in rhesus macaques while also generating T-cell immunity [69]. Specifically, ZIKA NS3 has been shown to activate CD8+ cytotoxic T lymphocytes (CTLs), providing protection against ZIKV challenge in mice [70]. In pregnant macaques, the ZIKV E protein induces CD4+ T cell responses but not CD8+ T cell responses [71]. Additionally, a previous study has shown that the ZIKV E protein primarily induces a Th2-type immune response, leading to the release of IFN-γ, IL-4, and IL-6 cytokines [72]. In the present study, we observed increased secretion of IL-2 and IL-6 in peripheral blood mononuclear cells (PBMCs) from rhesus macaques immunized with the EDIII-Fc vaccine compared to non-immunized macaques. IL-2 is known to enhance vaccine-mediated T-cell immunity [73], while IL-6 can induce T-cell proliferation and cytotoxic T-cell differentiation, playing a critical role in host defense against infectious microorganisms such as viruses and bacteria [74]. These findings indicate that the EDIII-Fc vaccine strategy can induce long-lasting memory T-cell immunity in rhesus macaques.

Indeed, the prevention and control of ZIKV requires a collaborative effort from individuals, communities, and global organizations. With the increase in international interactions, including travel and business activities in areas affected by ZIKV epidemics, the challenge of containing the spread of the disease has become more significant. Vaccination remains the most effective and safe approach for controlling highly pathogenic and infectious viral diseases like ZIKV.

Scientists around the world have been actively exploring different avenues for the development of Zika vaccines. Their dedicated research and efforts are aimed at providing effective preventive measures against this virus. We are hopeful that our own research will contribute to the global endeavor to combat the ZIKV, ultimately helping to reduce the impact of this disease and safeguard public health.

## Material and methods

### Ethics statement

The animal work in this study was conducted in compliance with the guidelines of and under a protocol approved by the Institutional Animal Care and Use Committee (IACUC) at the Institute of the Laboratory Animal Center, Southern Medical University (Protocol #L2016168). All applicable international, national, and/or institutional guidelines for the care and use of animals were followed.

### Cells

HEK 293T cells (ATCC CRL-11268) and Vero cells (ATCC CCL-81) were purchased from ATCC and cultured in DMEM medium (Gibco, USA) supplemented with 10% fetal bovine serum (FBS), 100 U/mL of penicillin (Gibco, USA), and 100 μg/mL of streptomycin (Gibco, USA).

### Viruses

The Asian lineage Z16006 strain (GenBank NO. KU955589.1) was obtained from the Institute of Microbiology in the Center for Disease Control and Prevention of Guangdong Province, China [75]. ZIKV was propagated in Vero cells at an MOI of 0.05. When cytopathic effects were observed, the supernatant was collected, centrifuged at 3000 rpm for 10 minutes, sterile filtered using a 0.45 μm syringe filter (Millipore, USA), and stored at -80°C.

### Construction of EDIII expression vectors

The E-DIII gene of the *Zika virus* Haiti2014 strain was selected from GenBank (Reference number KU509998.3) and synthesized by Sangon (China). The EDIII coding sequence with the human Ig1-Fc fragment was amplified by PCR and cloned into the lentiviral expression plasmid pLV-CMV-eGFP using NheI and MluI (NEB, UK).

### Recombinant lentivirus production

The lentiviral vectors were produced using the three-plasmid transfection system. HEK293T cells were seeded at a density of 5×10^6^ cells in 100 mm cell culture dishes and incubated at 37°C with 5% CO_2_. Transfection was performed when the cell density reached 70–80%. A total of 5 μg of the lentiviral expression plasmid pLV-ZIKV-EDIII, 3.75 μg of the lentiviral packaging plasmid psPAX2, and 1.25 μg of the lentiviral shuttle plasmid pMD2.G were added to 500 μL of 1× HBS buffer and thoroughly mixed. The plasmid mixture was then combined with 500 μL of PEI solution (2 μg/μL) and incubated at room temperature for 20 minutes. The resulting mixture was gently added dropwise to the cell culture supernatant in the 100 mm dish. The dish was gently shaken to ensure even distribution, and the cells were maintained at 37°C with 5% CO_2_. After 24 hours, GFP expression was observed using fluorescent microscopy to confirm successful transfection. The recombinant lentivirus was harvested from the cell supernatant at 80 hours post-transfection. The viral loads of the recombinant lentivirus were quantified using real-time PCR with the HIV qRT-PCR Titration Kit (GeneCopoeia, USA).

### Cell transduction in vitro and generation of EDIII-Fc protein-expressing clonal cell lines

A total of 1.5×10^5^ HEK 293 T cells /well were prepared in a 6-well plate. On the following day, cells in each well were infected with packaged recombinant lentivirus LVCMV- EDIII -eGFP at a MOI of 1000 (1000 viral genomes per cell) in DMEM medium containing 10% FBS with 6–8 μg/mL hexadimethrine bromide (Sigma, Germany), at 24 h after infection. The expression efficiency of EDIII protein were measured by Western blot using anti-Human Fc antibody. The infected cells were plated in three plates at a density of 0.8 cell/well in 100 μL of DMEM containing 10% FBS. Three weeks later, cell clones in good condition were picked and cultured.

### Protein purification and quantification

The recombinant cell line HEK293T-ZIKV-EDIII, which can stably secrete the expression of ZIKV EDIII protein, was passed through successive generations. The collected cell culture supernatants were centrifuged. Removing the cells and debris, the supernatant was filtered using a 0.45 μm syringe filters (Millipore, USA). EDIII-Fc protein was purified using protein-A affinity chromatography previously described [76]. BioLogic LP protein purifier was used for purification and quantification with a BCA protein quantification kit (Thermo, USA).

### Western blotting

20 μL of cell supernatant or cell lysate were added to 80 μL of 5× SDS loading buffer. The mixture was then boiled in a water bath for 10 minutes and subjected to 10% SDS-PAGE electrophoresis. After electrophoresis, the separation gel was transferred onto an NC membrane (Millipore, USA), and the membrane was blocked with 10% fat-free milk for 2 hours. Anti- ZIKV E protein polyclonal antibody (GeneTex, USA) was used and incubated overnight at 4°C. HRP-labeled goat anti-rabbit antibody (Abcam, USA) was used as the secondary antibody and incubated for 1 hour at 37°C. Finally, color development was performed using ECL chemiluminescence.

### Immunization of Balb/c mice with EDIII-Fc protein

Twenty-four 6-week-old female Balb/c mice were randomly divided into two groups, with 12 mice per group. All animal procedures were approved by the Animal Care and Use Review Committee at the Southern Medical University. To prepare the immunization solution, 4 mg of EDIII-Fc protein was dissolved in 100 mL of saline and thoroughly mixed with an equal volume of Freund’s complete adjuvant. The Balb/c mice were then immunized via abdominal subcutaneous multipoint injection, with each mouse receiving a total injection volume of approximately 0.25 mL. The control group was treated similarly, but with injections of the same volume of saline mixed with an equal volume of Freund’s complete adjuvant. After the initial immunization, the mice were given two additional booster injections. The first booster was administered in the second week following the initial immunization, and the second booster was given in the fourth week. To assess the immune response, blood samples were collected from the Balb/c mice by tail amputation. Pre-immune blood samples were obtained 2 days before immunization, and subsequent samples were collected on a weekly basis after the immunization. These blood samples were processed to obtain serum, which was then prepared for further analysis.

### Immunization of rhesus macaques with EDIII-Fc protein

Twelve rhesus macaques, each weighing approximately 6 kg, were selected. All animal procedures were approved by the Animal Care and Use Review Committee at the Southern Medical University. Blood was collected intravenously and tested for antibodies to Dengue virus and West Nile virus after excluding any history of infection with either of these viruses. The rhesus macaques were then randomly divided into two groups, with six macaques in each group.

To immunize the animals, a solution of 5 mg of EDIII-Fc protein was prepared by dissolving it in 100 mL of saline. The protein solution was then thoroughly mixed with an equal volume of MF59 adjuvant. The rhesus macaques were immunized via intramuscular injection in the thighs of their lower limbs, with each animal receiving a volume of 1 mL of the prepared mixture. The control group received the same volume of saline mixed with an equal volume of MF59 adjuvant, administered via the same route of administration. Additional immunizations were given once at week 4 and week 8 following the first immunization. Pre-immune blood samples were obtained 2 days before immunization, and subsequent samples were collected weekly from a vein after immunization. Serum was prepared from the collected blood samples.

### Enzyme-linked immunosorbent assay (ELISA)

To perform the ELISA, ZIKV E protein (Sino Biological, China), was diluted to a concentration of 5 μg/mL using a coating solution consisting of 0.05 M Na_2_CO_3_ at pH 9.0. Next, diluted EDIII-Fc protein (0.1 mL/well) was added to a 96-well plate and left to coat overnight at 4°C. Afterward, 250 μL of PBST solution with a final concentration of 10% fat-free milk was added to each well and incubated at 37°C for 2 hours. The serum samples, serially diluted at a ratio of 1:10, were added to the respective wells. For mouse serum, HRP-labeled sheep anti-mouse monoclonal antibody (diluted 1:100,000) was added and incubated at 37°C for 30 minutes. Similarly, for rhesus macaque serum, HRP-labeled mouse anti-monkey monoclonal antibody (diluted 1:50,000) was added and incubated at 37°C for 30 minutes. Following the incubation, each well was treated with 100 μL of TMB substrate for 30 minutes. The reaction was stopped by adding 100 μL of 2 M sulfuric acid after 15 minutes, with precautions taken to protect the samples from light. The optical density (OD) value was measured at 450 nm using a microplate reader.

### Plaque reduction neutralization test

To perform the plaque reduction neutralization test, the sera to be tested were serially diluted 2-fold in PBS starting from 1:5. The ZIKV was diluted with DMEM to a final concentration of 100 PFU/320 μL. Next, 320 μL of the virus dilution was mixed with 320 μL of serum dilution and incubated for 1 hour at 37°C. The virus-serum mixture was then inoculated into Vero cells cultured in 6-well plates and adsorbed for 1 hour at 37°C. After discarding the virus-serum mixture, the cells were washed once with PBS, and 2 mL of 37°C nutrient agar was added. The plates were then incubated at 37°C for 3 days. Once etiolated spots were observed microscopically, 2 mL of 4% formaldehyde was added to each well to fix the nutrient agar at room temperature for 30 minutes. The fixed nutrient agar was washed off with deionized water, and 1 mL of 1.5% crystalline violet was added and allowed to stand for 10 minutes. The crystalline violet was then discarded, and the cells were washed twice with deionized water. The results are reported as the fold dilution of serum that inhibited 50% of plaque formation.

### ZIKV challenge studies

For the ZIKV challenge studies, following the previously described protocol [29], seven Balb/c mice were subcutaneously injected with 10^4^ PFU of ZIKV in the foot on the 7th day after the second immunization, which took place in the P3 laboratory. Daily blood samples were collected from the tail vein of the mice following the challenge to detect the viral load in the serum.

### qPCR for mouse serum viral load detection

Mouse serum viral load was detected using qPCR. RNA was extracted from mouse serum using a viral RNA extraction kit (Tiangen, China). The primers of ZIKV NS5 used for qPCR were as follows: Forward primer: 5′-GGC RTT RGC CAT CAG TCG-3′, Reverse primer: 5′-ATG GAG CAT CAT CCG KGAG ACT-3′ [77]. A fluorescent quantitative PCR kit (QIAGEN, Germany) was used for virus quantification in the serum.

### Th1/Th2/Th17 cytokines analysis

Before immunization, 8 mL of blood was collected intravenously in sterile tubes containing anticoagulant, as well as at the 4th, 8th, and 12th week thereafter. PBMC layers were obtained by gradient centrifugation using Lymphoprep (STEMCELL, Canada) after dilution with an equal volume of separation buffer (consisting of 0.05 M PBS, 2 mM EDTA, pH = 7.2). Following two washes with 0.05 M PBS (pH = 7.2), the cells were counted and diluted to a concentration of 108 cells/mL using 1640 basal medium (Gibco, USA). Subsequently, 10^7^ PBMC cells were inoculated into 10 mm cell culture dishes and incubated for 48 hours with a final concentration of 20 μg/mL of ZIKV E protein (Sino Biological). Culture supernatants were collected, and flow cytometric assays were performed according to the instructions provided with the Th1/Th2/Th17 Cytokine Assay Kit (BD Biosciences, USA).

### Statistical analysis

The statistical analysis was performed using GraphPad Prism software version 9.0 (San Diego, USA). For comparisons between two normally distributed groups, an unpaired t-test was employed. When analyzing more than two groups, a Kruskal-Wallis test with Dunn’s multiple comparison post-test was conducted. Additionally, normally distributed data were analyzed using a one-way analysis of variance (ANOVA) with Bonferroni’s multiple comparison post-test. The data are presented as mean ± SEM. Significance levels were denoted as follows: * for *P* < 0.05, ** for *P* < 0.01, *** for *P* < 0.001, and **** for *P* < 0.0001, indicating increasing levels of significance.

## Supporting information

S1 Fig The ZIKV EDIII nucleotide sequence alignment. Sequence alignments were generated using NCBI Multiple Alignment Viewer (https://www.ncbi.nlm.nih.gov/projects/msaviewer/). A total of 1,809 EDIII protein sequences data were obtained from Genbank, and the date was to June 20, 2023).
(PDF)
