# Supplementary material for: EDIII-Fc induces protective immune responses against the Zika virus in mice and rhesus macaque
Source: PLoS Negl Trop Dis. 2023 Nov 20;17(11):e0011770. doi: 10.1371/journal.pntd.0011770 (PMC10695381; doi:10.1371/journal.pntd.0011770)
Supplement: S1 Fig — Sequence alignments were generated using NCBI Multiple Alignment Viewer (https://www.ncbi.nlm.nih.gov/projects/msaviewer/). A total of 1,809 EDIII protein sequences data were obtained from Genbank, and the date was to June 20, 2023). (PDF) [file pntd.0011770.s001.pdf]

NCBI Multiple Sequence Alignment Viewer, Version 1.21.0

Sequence ID	Start	Alignment	End	Organism	Date	Host	Source	Gene	Identity	Coverage	Mismatches
		1 10 20 30 40 50 60 70 80 90 100 109									
Query_2501 (+)	1		109						100.00	100.00	0
QDP14363.1 (+)	63		171	Zika virus	07-Mar-...	Aedes sp.			100.00	100.00	0
5KVD_E (+)	2		110	Zika virus					100.00	100.00	0
5OMZ_A (+)	5		113	Zika virus					100.00	100.00	0
QDP14362.1 (+)	64		172	Zika virus	07-Mar-...	Aedes sp.			100.00	100.00	0
QDP14366.1 (+)	64		172	Zika virus	07-Mar-...	Aedes sp.			100.00	100.00	0
QDP14367.1 (+)	64		172	Zika virus	07-Mar-...	Aedes sp.			100.00	100.00	0
5GZN_A (+)	299		407	Zika virus					100.00	100.00	0
5JHL_A (+)	300		408	Zika virus					100.00	100.00	0
7BQ5_A (+)	299		407	Zika virus					100.00	100.00	0
7BPK_A (+)	300		408	Zika virus					100.00	100.00	0
APO40589.1 (+)	284		392	Zika virus	Jan-2016	Aedes a...			100.00	100.00	0
AVK43536.1 (+)	299		407	Zika virus	Jan-2016	Homo s...			100.00	100.00	0
AWW21438.1 (+)	288		396	Zika virus	10-Nov-...	Homo s...			100.00	100.00	0
AWW21435.1 (+)	288		396	Zika virus	10-Nov-...	Homo s...			100.00	100.00	0
AZS35397.1 (+)	285		393	Zika virus	2017	Homo s...	serum		100.00	100.00	0
AZS35400.1 (+)	285		393	Zika virus	2017	Homo s...	serum		100.00	100.00	0
AWW21437.1 (+)	288		396	Zika virus	10-Nov-...	Homo s...			100.00	100.00	0
AWW21434.1 (+)	288		396	Zika virus	10-Nov-...	Homo s...			100.00	100.00	0
AZS35396.1 (+)	290		398	Zika virus	2017	Homo s...	serum		100.00	100.00	0
ASN64426.1 (+)	291		399	Zika virus	Jan-2013	Homo s...	plasma		100.00	100.00	0
AQQ13230.1 (+)	1		105	Zika virus	24-Feb-...	Homo s...			99.05	96.33	1
ASN64427.1 (+)	299		407	Zika virus	Jun-2013	Homo s...	plasma		100.00	100.00	0
ANF28857.1 (+)	299		407	Zika virus	2015	Homo s...			100.00	100.00	0
YP_009430300.1 (+)	299		407	Zika virus	2015	Homo s...	fetus' bra...	POLY	100.00	100.00	0
ARO85704.1 (+)	299		407	Zika virus	10-Oct-2...	Homo s...	culture		100.00	100.00	0
AWW21436.1 (+)	288		396	Zika virus	10-Nov-...	Homo s...			100.00	100.00	0
QCQ29072.1 (+)	300		408	Zika virus	25-Sep-...	Aedes a...			100.00	100.00	0
6JFH_A (+)	299		407	Zika virus					100.00	100.00	0
ATS91863.1 (+)	299		407	Zika virus	11-Jul-2...	Homo s...	saliva		100.00	100.00	0
5LBS_A (+)	299		407	Zika virus					100.00	100.00	0
AQQ13229.1 (+)	1		105	Zika virus	19-Feb-...	Homo s...			99.05	96.33	1
AOT82811.1 (+)	304		412	Zika virus	16-May-...	Homo s...	serum		100.00	100.00	0
ANF28861.1 (+)	299		407	Zika virus	2016	Homo s...			100.00	100.00	0
ANF28862.1 (+)	299		407	Zika virus	2016	Homo s...			100.00	100.00	0
ARO85706.1 (+)	299		407	Zika virus	05-Mar-...	Homo s...	culture		100.00	100.00	0
ANK57898.1 (+)	299		407	Zika virus	2016	Homo s...			100.00	100.00	0
AWW21433.1 (+)	288		396	Zika virus	10-Nov-...	Homo s...			99.08	100.00	1
AVV61952.1 (+)	297		405	Zika virus	16-Apr-2...	Homo s...			100.00	100.00	0
AWW21432.1 (+)	288		396	Zika virus	10-Nov-...	Homo s...			99.08	100.00	1
AWW21431.1 (+)	288		396	Zika virus	10-Nov-...	Homo s...			99.08	100.00	1
AZS35398.1 (+)	285		393	Zika virus	2017	Homo s...	serum		99.08	100.00	1
AQU11806.1 (+)	249		357	Zika virus	1989	mosquito			98.17	100.00	2
AZS35402.1 (+)	285		393	Zika virus	2017	Homo s...	serum		99.08	100.00	1
AVV61891.1 (+)	299		407	Zika virus	28-Feb-...	Homo s...	saliva		99.08	100.00	1
ATW74960.1 (+)	299		407	Zika virus	11-Aug-...	Homo s...	serum		99.08	100.00	1
QCH40598.1 (+)	289		397	Zika virus	2018-06	Anophel...			98.17	100.00	2
QCH40599.1 (+)	289		397	Zika virus	2018-06	Anophel...			98.17	100.00	2
6NIP_E (+)	299		405	Zika virus ZIKV/H. sapie...					100.00	98.17	0
ALX35662.1 (+)	303		411	Zika virus	Oct-2015	Homo s...	serum		100.00	100.00	0
7JYI_C (+)	299		407	Zika virus					97.25	100.00	3
ATW74961.1 (+)	299		407	Zika virus	27-Jun-2...	Homo s...	serum		98.17	100.00	2
6JEP_E (+)	1		101	Zika virus					100.00	92.66	0
ATS91865.1 (+)	299		407	Zika virus	26-May-...	Homo s...	serum		99.08	100.00	1
AOX24134.1 (+)	389		497	Zika virus	01-Sep-...	Homo s...	urine		99.08	100.00	1
AXE75585.1 (+)	299		407	Zika virus	21-Oct-2...	Homo s...			98.17	100.00	2
AOX24135.1 (+)	491		599	Zika virus	30-Aug-...	Homo s...	urine		100.00	100.00	0
YP_009227198.1 (+)	295		403	Zika virus		sentinel...		POLY	96.33	100.00	4
ASV60828.1 (+)	299		407	Zika virus	11-Nov-...	Homo s...			96.33	100.00	4
ASV60826.1 (+)	299		407	Zika virus	05-Jul-2...	Homo s...			96.33	100.00	4
5H32_A (+)	299		403	Zika virus					100.00	96.33	0
QGW51946.1 (+)	589		697	Zika virus	Dec-2015	Homo s...			100.00	100.00	0
APG56497.1 (+)	589		697	Zika virus	Oct-2016	Homo s...			100.00	100.00	0
APG56494.1 (+)	589		697	Zika virus	Aug-2016	Homo s...			100.00	100.00	0
ASV60827.1 (+)	299		407	Zika virus	10-Jul-2...	Homo s...			96.33	100.00	4
APG56493.1 (+)	589		697	Zika virus	Jun-2016	Homo s...			100.00	100.00	0
QGW51952.1 (+)	589		697	Zika virus	19-Feb-...	Homo s...			100.00	100.00	0
APG56495.1 (+)	589		697	Zika virus	Sep-2016	Homo s...			100.00	100.00	0
QGW51947.1 (+)	589		697	Zika virus	2010	Homo s...			100.00	100.00	0
QGW51949.1 (+)	589		697	Zika virus	Dec-2015	Homo s...			99.08	100.00	1
APG56496.1 (+)	589		697	Zika virus	Oct-2016	Homo s...			99.08	100.00	1
QGW51945.1 (+)	589		697	Zika virus	2013	Homo s...			99.08	100.00	1
AML81023.1 (+)	589		697	Zika virus	Jan-2016	Homo s...	serum		100.00	100.00	0
AML81022.1 (+)	589		697	Zika virus	Dec-2015	Homo s...	serum		100.00	100.00	0
ALX35660.1 (+)	589		697	Zika virus	Oct-2015	Homo s...	serum		100.00	100.00	0
AML81021.1 (+)	589		697	Zika virus	Dec-2015	Homo s...	serum		100.00	100.00	0
AML81026.1 (+)	589		697	Zika virus	Jan-2016	Homo s...	serum		100.00	100.00	0
AML81025.1 (+)	589		697	Zika virus	Jan-2016	Homo s...	serum		100.00	100.00	0
QGW51942.1 (+)	589		697	Zika virus	17-Nov-...	Homo s...			98.17	100.00	2
AML81019.1 (+)	589		697	Zika virus	Jan-2016	Homo s...	urine		100.00	100.00	0
AML81020.1 (+)	589		697	Zika virus	Jan-2016	Homo s...	serum		100.00	100.00	0
AML81027.1 (+)	589		697	Zika virus	Dec-2015	Homo s...	serum		100.00	100.00	0
AOX49268.1 (+)	589		697	Zika virus	Apr-2016	Homo s...	semen		100.00	100.00	0
AOY10606.1 (+)	589		697	Zika virus	Apr-2016	Homo s...	saliva		100.00	100.00	0
AMC37200.1 (+)	299		407	Zika virus	10-Nov-...	Homo s...			100.00	100.00	0
AOX49267.1 (+)	589		697	Zika virus	Jun-2016	Homo s...	urine		100.00	100.00	0
QUJ10646.1 (+)	331		439	Zika virus	24-Jan-2...	Homo s...			100.00	100.00	0
AZS35391.1 (+)	589		697	Zika virus	07-Apr-2...	Homo s...	urine		100.00	100.00	0
ARB07955.1 (+)	501		609	Zika virus	01-Aug-...	Homo s...	serum		100.00	100.00	0
AXE75583.1 (+)	299		407	Zika virus	01-Apr-2...	Homo s...			96.33	100.00	4
AZS35366.1 (+)	589		697	Zika virus	10-Feb-...	Homo s...	serum		100.00	100.00	0
AQS26817.1 (+)	589		697	Zika virus	25-Apr-2...	Homo s...			100.00	100.00	0
AQS26830.1 (+)	505		613	Zika virus	Jan-2016	Homo s...			100.00	100.00	0
AZS35369.1 (+)	589		697	Zika virus	20-Jan-2...	Homo s...	serum		100.00	100.00	0
AZU90721.1 (+)	589		697	Zika virus	05-Aug-...	Homo s...	urine		100.00	100.00	0
AZS35340.1 (+)	589		697	Zika virus	06-Apr-2...	Homo s...	saliva		100.00	100.00	0
AZS35350.1 (+)	589		697	Zika virus	15-Feb-...	Homo s...	plasma		100.00	100.00	0
AQS26823.1 (+)	589		697	Zika virus	13-Mar-...	Homo s...			100.00	100.00	0
AZS35385.1 (+)	589		697	Zika virus	24-Mar-...	Homo s...	plasma		100.00	100.00	0
AZS35368.1 (+)	589		697	Zika virus	25-Jan-2...	Homo s...	urine		100.00	100.00	0
AZS35373.1 (+)	589		697	Zika virus	25-Jan-2...	Homo s...	serum		100.00	100.00	0
